# Effect of P21‐activated kinase 1 (PAK‐1) inhibition on cancer cell growth, migration, and invasion

**DOI:** 10.1002/prp2.518

**Published:** 2019-09-06

**Authors:** Wided Najahi‐Missaoui, Nhat D. Quach, Amber Jenkins, Isha Dabke, Payaningal R. Somanath, Brian S. Cummings

**Affiliations:** ^1^ Department of Pharmaceutical and Biomedical Sciences, College of Pharmacy University of Georgia Athens GA USA; ^2^ Clinical and Experimental Therapeutics, College of Pharmacy University of Georgia and Charlie Norwood VA Medical Center Augusta GA USA; ^3^ Department of Medicine, Vascular Biology Center and Cancer Center Georgia Regents University Augusta GA USA; ^4^ Interdisciplinary Toxicology Program University of Georgia Athens GA USA; ^5^ Present address: Department of Molecular Pharmacology, Physiology, & Biotechnology Brown University Providence RI USA; ^6^ Present address: Cancer Center of Middle Georgia Dublin GA USA; ^7^ Present address: Medical College of Georgia Augusta GA USA

**Keywords:** breast cancer, in vitro, P21‐activated kinase, prostate cancer

## Abstract

P21‐activated kinase‐1 (PAK‐1) is a serine/threonine kinase involved in multiple signaling pathways that mediate cellular functions such as cytoskeletal motility, cell proliferation, and survival. PAK‐1 expression is altered in various cancers, including prostate and breast. Our recent studies showed that prostate cancer cells expressing higher levels of PAK‐1 were resistant to the cytotoxic effects of the PAK‐1 inhibitor, inhibitor targeting PAK‐1 activation‐3 (IPA‐3), compared to those with lower expression. This study expanded these findings to other cancers (breast and melanoma) by testing the hypothesis that genetic and pharmacological inhibition of PAK‐1 alters cell growth, migration, and invasion in prostate, breast, and skin cancer cell lines. We also tested the specificity of IPA‐3 for PAK‐1 and the hypothesis that gene silencing of PAK‐1 altered the efficacy of sterically stabilized liposomes (SSL) containing IPA‐3 (SSL‐IPA‐3). PAK‐1 expression was identified in four different breast cancer cell lines, and in a melanoma cell line. The expression of PAK‐1 correlated to the IC_50_ of IPA‐3 as measured by MTT staining. PAK‐1 inhibition using shRNA correlated with decreased cell migration and invasion in prostate cancer DU‐145 and breast cancer MCF‐7 cells. Decreased migration and invasion also correlated to decreased expression of E‐cadherin and alterations in C‐X‐C Chemokine Receptor type 4 and Homing Cell Adhesion Molecule expression. PAK‐1 inhibition increased the cytotoxicity of IPA‐3, and the cytotoxicity of SSL‐IPA‐3 to levels comparable to that of free drug. These data demonstrate that both pharmacological and molecular inhibition of PAK‐1 decreased growth in prostate, breast, and melanoma cancer cell lines, and increased the toxicity of IPA‐3 and its liposomal formulation. These data also show the specificity of IPA‐3 for PAK‐1, are some of the first data suggesting that IPA‐3 is a therapeutic treatment for breast cancer and melanoma, and demonstrate the efficacy of liposome‐encapsulated IPA‐3 in breast cancer cells.

**Abbreviations**
CXCR‐4: C‐X‐C Chemokine Receptor type 4
DAPI: 4′,6‐diamidino‐2‐phenylindole
EMT: epithelial‐mesenchymal transition
HCAM: homing cell adhesion molecule
IPA‐3: Inhibitor targeting PAK‐1 activation‐3
KD: knockdown
MTT: 3‐(4, 5‐dimethylthiazol‐2‐yl)‐2, 5‐diphenyltetrazolium bromide
PAK‐1: P21‐activated kinase 1
PBS: phosphate saline buffer
SSL: sterically stabilized liposomes
TGFβ: transforming growth factor‐β

## 1 INTRODUCTION

P21‐activated kinases (PAKs) are serine/threonine kinases that mediate multiple signal transduction pathways, including those that control cellular functions such as cytoskeletal motility, cell proliferation, and survival.^1^, ^2^ Upregulation of PAKs in tumors is suggested to increase cellular transformation, motility, and invasion in surrounding tissues leading to cancer metastasis.^3^, ^4^ In addition, overactivation of PAKs results in downregulation of proapoptotic pathways and promotion of cell survival.^3^ While it has been reported that PAK‐6, a group II PAK, is overexpressed in prostate cancer,^5^, ^6^ recent studies from our laboratory and others have shown that PAK‐1 is also overexpressed in prostate tumor tissues and in metastasized sites in the human lung.^2^, ^7^ PAK‐1 is a major downstream effector of Rac1, which mediates cytoskeletal remodeling during prostate cancer invasion.^8^, ^9^, ^10^

Previous studies showed that PAK‐1 mediated the growth of prostate PC‐3 cell tumor xenografts in athymic nude mice as well as the transforming growth factor‐β (TGFβ)‐induced prostate cancer cell epithelial‐mesenchymal transition (EMT).^11^ These studies suggested that PAK‐1 plays a major role in prostate cancer progression and is a potential target for prostate cancer therapy. PAK‐1 has also been suggested to be involved in the early stages of breast cancer and may partially participate in the mechanisms mediating the transformation of mammary epithelial cells into mesenchymal malignant cells.^12^ Studies have also shown that overexpressed or hyperactivated PAK‐1 mediates the anchorage independence of transformed epithelial cells during the progression of breast cancer.^12^, ^13^ PAK‐1 is also essential for AKT‐ and Ras‐induced oncogenic transformations in both prostate and breast cancer cells.^14^, ^15^

In addition to their catalytic active site, most PAKs, including PAK‐1, have critical conformations that are required for their functions. These critical conformational sites may prove useful for the design of allosteric small molecule inhibitors whose efficacy do not depend on targeting the catalytic site of PAKs, which are ATP‐binding domain common to many kinases.^16^ Such precision targeting may lower off‐target toxicity and increase specificity. This hypothesis is supported by data derived from an allosteric small molecule inhibitor of group I PAKs called “inhibitor targeting PAK‐1 activation‐3” (IPA‐3).^17^ We provided further support for this hypothesis by showing that IPA‐3 decreased prostate tumor growth *in vitro* and *in vivo*.^11^^,^ 18

Despite the promising effect of IPA‐3 on prostate tumor growth, this compound has some drawbacks that limit its pharmacological potential. The primary one of these is that IPA‐3 is metabolically unstable and required daily injections for its efficacy.^11^ We addressed this limitation by developing a novel liposomal formulation of IPA‐3 using sterically stabilized liposomes (SSL‐IPA‐3) and determined that these nanoparticles decreased prostate cancer tumor growth in vivo as compared to free IPA‐3, with less frequent dosing (every 3 days).^18^ Our data also showed the novel finding that both free IPA‐3 and that encapsulated in SSL decreased the viability of breast cancer cells.^18^ Not surprisingly, the efficacy of both free IPA‐3 and SSL‐IPA‐3 correlated to PAK‐1 expression in that IPA‐3 was more efficacious at limiting viability in cells with lower levels of PAK‐1 compared to high PAK‐1 expressing cells. This raised concerns whether the efficacy of IPA‐3 was truly dependent on PAK‐1 expression.

This study used both pharmacological and molecular approaches to investigate the hypothesis that the efficacy of free IPA‐3 and SSL‐IPA‐3 is dependent on the expression of PAK‐1 in diverse cancer cells, including prostate and breast cancer, as well as melanoma cells. These studies showed that cancer cells with high PAK‐1 expression were less responsive to the cytotoxicity of IPA‐3 and SSL‐IPA‐3 compared to cells with low PAK‐1 expression. This current study also investigated the ability of PAK‐1 gene silencing to alter prostate and breast cancer cell growth and alter markers of EMT. These data demonstrate that the toxicity of IPA‐3 is partially mediated by PAK‐1 expression, and support the clinical potential of SSL‐IPA‐3 for the treatment of cancers with altered PAK‐1 expression.

## 2 MATERIALS AND METHODS

### 2.1 Cell lines and cell culture

The human breast cancer cell lines BT‐474, MCF‐7, MDA‐231, MDA‐468, the melanoma‐derived cell line, MDA‐435, and the immortalized breast epithelial cell line, MCF‐10A, were purchased from ATCC. MCF‐10A cells were grown in F12/DMEM (50/50) medium, and the rest of the cells were all cultured in RPMI medium. All culture media were supplemented with 10% (v/v) fetal bovine serum (FBS) and 1% (v/v) penicillin/streptomycin antibiotics (ATCC). The human prostate cancer cell line, DU‐145, was purchased from ATCC, and was also maintained in RPMI medium supplemented with 10% FBS and 1% penicillin/streptomycin. All the cells were maintained in a humidified atmosphere at 37°C in incubators with 5% CO_2_. The DU‐145 cells were chosen based on our previous studies that showed the cells to be resistant to the activity of both free and encapsulated IPA‐3.^18^

### 2.2 Chemicals and reagents

IPA‐3 was purchased from Tocris Bioscience. The phospholipids to prepare liposomes were purchased from Avanti Polar Lipids, Inc. Cholesterol and MTT [3‐(4, 5‐dimethylthiazol‐2‐yl)‐2, 5‐diphenyltetrazolium bromide] were purchased from Sigma‐Aldrich. RPMI cell culture media and their supplements, including antibiotics and FBS, were purchased from ATCC. Mission shRNA lentiviral transduction particles (SHCLNV) and control shRNA lentiviral particles (SHC002V) were obtained from Sigma‐Aldrich. The annexin V/PI detection kit was purchased from Fisher Scientific. The phospholipids, 1, 2‐distearoyl‐*sn‐*glycero‐3‐phosphatidylcholine (DSPC), and 1, 2‐distearoyl‐*sn‐*glycero‐3‐phosphoethanolamine–*N*‐poly (ethylene glycol) 2000 (DSPE‐PEG), were purchased from Avanti Polar Lipids, Inc. All other chemicals and solvents were of analytical grade and were obtained from Sigma Aldrich or Fisher Scientific.

### 2.3 Lentiviral transduction

The transduction of PAK‐1 knockdown (KD) and control lentiviral particles was performed according to the manufacturer's instructions. Briefly, cells (DU‐145 and MCF‐7) were plated in 96‐well plates in RPMI medium for 24 hours prior to transduction. Cells were treated with the lentiviral particles and then incubated overnight at 37°C. The transduced cells were maintained in RPMI^®^1640 medium with 2 μg/mL of puromycin (Sigma Aldrich Inc). Media were replaced every 3‐4 days with fresh, puromycin‐containing media until resistant colonies were identified. The efficiency of the shRNA lentiviral particles targeting PAK‐1 was measured by assessing protein expression using immunoblot analysis. Stable KD clones and control clones (those transduced with control particles) were named PAK‐1 knockdown (PAK‐1 KD) and PAK‐1 control, respectively, for both DU‐145 and MCF‐7 cell lines.

### 2.4 Immunoblot analysis

Cell lysates from different cell lines were collected in RIPA buffer, which contained a protease inhibitor cocktail (Santa Cruz Biotechnology, Inc). The BCA assay was used to determine protein concentrations. Samples of 40 µg of protein were separated using SDS‐PAGE and then transferred to nitrocellulose membranes that were then blocked in 5% (w/v) nonfat dry milk in Tris‐buffered saline‐Tween 20 (TBST). After 2 hours of blocking, the membranes were incubated with a rabbit PAK‐1 antibody (Cell Signaling Technology) at a dilution of 1:1000 in 1% (w/v) BSA TBST overnight. The antibody against GAPDH (Santa Cruz Biotechnology Inc) was used at a dilution of 1:4000 in 1% (w/v) BSA in TBST for 1 hour. Membranes were then incubated with the appropriate peroxidase‐conjugated secondary antibodies (Promega,) used at a dilution of 1:2500. The membranes were then washed with TBST three times for 10 minutes each. Bands were visualized using chemiluminescent substrates (Thermo Scientific) and imaged with a FluorChem SP digital Imager (Alpha Innotech). Immunoblot analysis was performed on protein samples from at least three different passages (n = 3) of control and KD cells. Densitometry was performed using National Institutes of Health Image J software.

### 2.5 Determination of cell growth

Cell growth was measured using crystal violet staining. DU‐145 and MCF‐7 cells were seeded in 4000 and 10 000 cells, respectively, into each well of 6‐well plates in triplicate. Cells were maintained at 37°C for 10 days and media were changed every 3 days. Cells were fixed with 4% formaldehyde for 20 minutes and then stained using 1% (v/v) crystal violet at room temperature for 20 minutes. Excess crystal violet staining solution was then removed and the plates were washed with water and left to air dry at room temperature. Cells were then imaged using a Canon EOS Rebel T3i camera.

### 2.6 Measurement of cell migration

Cells were seeded in 6‐well plates at a density of 1 × 10^6^ cells/well under the above conditions. Cell migration was assessed once cells reached a confluency of at least 90% (after 48 hours) using the scratch wound healing assay as previously described.^19^ Briefly, a “wound gap” was created in the cell monolayer by scratching using 1‐mL pipette tips. The migration ability of the different cells was monitored, imaged, and quantified after 24‐72 hours. Cells were fixed with 4% (v/v) formaldehyde for 20 minutes followed by 20 minutes staining with 1% (v/v) crystal violet in PBS. The excess of crystal violet was washed with water three times. Plates were left to air dry and then imaged using an inverted fluorescence microscope equipped with an AxioCam MRc5 digital camera (Carl Zeiss MicroImaging Inc). The gaps from the scratch were imaged, measured, and normalized to the calculated control wound closure.

### 2.7 Measurement of cell invasion

The 8‐µm Transwell^®^ invasion plates were prepared by coating the top chamber with 100‐µL Matrigel^®^ (in serum‐free media). Culture media (750 µL) with 10% FBS were added to the lower chamber (chemoattractant compartment). Cells (100 µL of 5 × 10^5^ cells) in serum‐free media were added to the top chamber. Plates were then incubated at 37°C for 24 hours (for DU‐145 cells) and 72 hours (for MCF‐7 cells). The different times were used due to differences in intrinsic cell migration ability of each cell line. Media were removed after the indicated times and remaining cells were scraped off from the top chamber. The chambers were washed twice with PBS and cells were then fixed using 4% (v/v) formaldehyde at room temperature for 20 minutes. The formaldehyde was then removed and the cells were washed twice with PBS. Cells were permeabilized with 100% methanol at room temperature for 20 minutes and then stained with 4′, 6‐diamidino‐2‐phenylindole (DAPI) at room temperature for 20 minutes, after which the cells were washed and imaged using an inverted fluorescence microscope equipped with an AxioCam MRc5 digital camera (Carl Zeiss MicroImaging Inc). Quantification of invasive cells was accomplished by counting cells in at least three different fields.

### 2.8 Preparation of sterically stabilized IPA‐3 liposomes

Sterically stabilized IPA‐3 (SSL‐IPA‐3) liposomes were prepared as described in our previous study^18^ using the thin lipid hydration method followed by freeze‐thaw cycles and a high‐pressure extrusion.^20^, ^21^ Our previous study also described the physical characteristics, stability, and composition of these liposomes.^18^ Briefly, cholesterol (5 µmol/mL), phospholipids, including DSPC (9 µmol/mL) and DSPE‐PEG (1 µmol/mL) in chloroform, and IPA‐3 (4 µmol/mL) in ethanol were added into a round bottom flask, the solvents were then evaporated under vacuum in a water bath at 65°C using a rotary evaporator (Buchi Labortechnik AG). The formed thin film was then hydrated and suspended in PBS to achieve a final lipid concentration of 10 µmol/mL. The formulation then underwent five liquid nitrogen freeze‐thaw cycles above the phase transition temperature of the primary lipid, prior to passing five times through a Lipex extruder (Northern Lipids, Inc) at 65°C using double stacked polycarbonate membranes (80 nm, GE Osmonics). Excess unencapsulated IPA‐3 and lipids were eliminated using dialysis in 10% (w/v) sucrose for at least 20 hours with three changings of the dialysis media. Liposome suspensions were stored at 4°C, protected from light, and used within 24‐48 hours of preparation. Empty SSL (made without IPA‐3 encapsulation) were also formulated and used as vehicle controls. Quantification of IPA‐3 was evaluated using methods previously described by us.^18^

### 2.9 MTT staining and cell viability

The MTT assay was used to determine the IC_50_ of free IPA‐3 in breast and melanoma cells, following treatment with free and liposomal IPA‐3 (SSL‐IPA‐3) in the PAK‐1 KD cells (DU‐145 and MCF‐7) and their respective controls.^22^ Cells were seeded in 48‐well tissue culture plates at 5 x 10^4^ cells/ml and incubated at 37°C in a 5% CO_2_ incubator for 24 hours to allow the cells to attach and grow. Liposomes were diluted in culture media to their final concentrations and all experiments were performed in triplicate. Cells were also treated with DMSO (vehicle control for free IPA‐3) and empty liposomes (vehicle control for encapsulated IPA‐3). The cells were incubated for 24, 48, and 72 hours. MTT was added at each time point, at a final concentration of 0.25 mg/mL, and plates were incubated at 37°C for 2 hours. Nonreduced MTT and media were then aspirated and replaced with DMSO to dissolve the MTT formazan crystals. Plates were shaken for additional 15 minutes and absorbance was read at 590 nm using a Spectra Max M2 plate reader (BMG Lab Technologies, Inc).

### 2.10 Assessment of annexin V and propidium iodide staining

Cells in which PAK‐1 was inhibited were exposed to free IPA‐3 and SSL‐IPA‐3 dosing for 48 hours. This was followed by assessment of annexin V‐FITC (marker of apoptosis) and propidium iodide (PI, marker of necrosis) staining using flow cytometry. DU‐145 PAK‐1 KD and MCF‐7 PAK‐1 KD cells were seeded and allowed to grow for 24 hours prior to treatment with free IPA‐3, SSL‐IPA‐3, and the controls of DMSO or empty liposomes. Cells were collected and then stained according to the manufacturer protocol using the FITC annexin V apoptosis detection kit (Fisher Scientific). Staining was quantified using a Dako Cyan flow cytometer. For each measurement, 20 000 events were counted. The different populations corresponding to viable and non‐apoptotic (annexin V‐PI‐), apoptotic (annexin V+PI‐), and late apoptotic (annexin V+PI+) cells, as well as necrotic cells (annexin V‐PI+) (Q4‐Q1, respectively) were shown using the plots of annexin V FITC vs PI from gated cells.

### 2.11 Statistical analysis

All experiments were repeated at least three times (n = 3). Results are shown as the average of all replicates ± SEM. An unpaired two‐tailed Student's *t* test was used to compare data sets with normal distribution. A nonparametric test such as the Mann‐Whitney test was used if data did not have Gaussian distribution using GraphPad Prism software. The significance level (alpha) was set at .05 (marked with symbols (*) wherever differences are statistically significant).

## 3 RESULTS

### 3.1 Correlation between PAK‐1 protein expression and IPA‐3 efficacy

**Figure 1:**
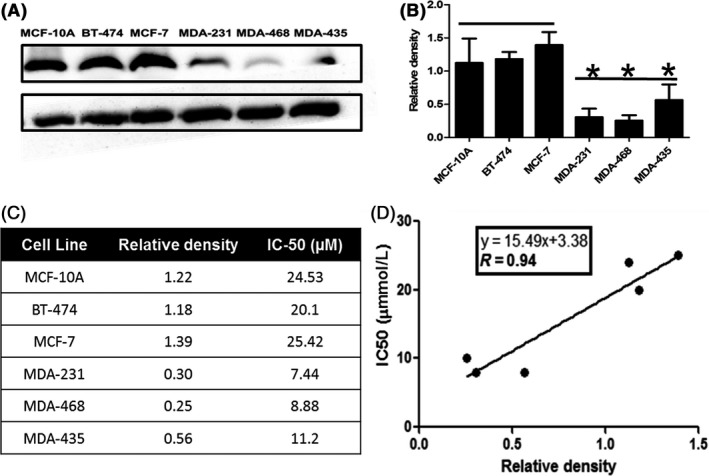
Expression of PAK‐1 and efficacy of IPA‐3 in breast cancer and melanoma cells. (A) Expression of PAK‐1 in breast cancer and melanoma cell lines as determined using immunoblot analysis. (B) Densitometry analysis of PAK‐1 expression. (C) IC_50_^’^s of IPA‐3 and relative density of PAK‐1 cancer cell lines. (D) Correlation between PAK‐1 expression and the IC_50_ (µM) of the PAK‐1 inhibitor IPA‐3. Data are representative of three different experiments using three different passages (n = 3). Data are presented as the mean ± SEM; *Indicates a significant (*P* < .05) difference as compared to the highest PAK‐1‐expressing cells (MCF‐7)

Only a few studies exist examining the effect of PAK‐1 inhibition on breast cancer cell growth and none could be found on PAK‐1 expression or inhibition in melanoma. As such, the expression of PAK‐1 in cell lines derived from noncancerous breast (MCF‐10A), breast cancer (BT‐474, MCF‐7, MDA‐321, MDA‐468), and melanoma (MDA‐435) was determined using immunoblot analysis (Figure 1). The data showed differential PAK‐1 expression across all cell lines, with PAK‐1 expression being higher in cell lines derived from noncancerous or earlier stage breast cancer (Figure 1A,B). In contrast, PAK‐1 expression was significantly lower in metastatic and triple‐negative breast cancer cell lines (MDA‐231, MDA‐468). PAK‐1 expression was also relatively lower in MDA‐435 cells, which are a melanoma‐derived cell line. These cells were treated with free IPA‐3 (Supplemental Figure S1) and IC_50_ values were estimated from the dose‐response curves (Figure 1C,D). There was an excellent correlation between the expression of PAK‐1 and the IC_50_ of free IPA‐3 (Figure 1D).

### 3.2 Transduction of shRNA decreased PAK‐1 expression in DU‐145 and MCF‐7 cells

**Figure 2:**
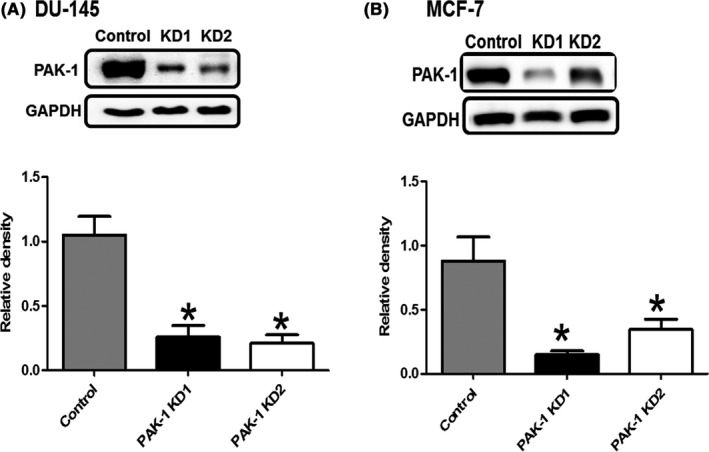
Effect of PAK‐1 shRNA on its expression in prostate and breast cancer cells. Expression of PAK‐1 in DU‐145 PAK‐1 KD (A) and MCF‐7 PAK‐1 KD (B) compared to their respective controls as determined by immunoblot analysis. Histograms are showing densitometry analysis of PAK‐1 expression in the immunoblots. Data are representative of three different experiments using three different passages (n = 3). Data are presented as the mean ± SEM; *Indicates a significant (*P* < .05) difference as compared to control cells

We chose to study the effect of PAK‐1 molecular inhibition on prostate and breast cancer cell growth using DU‐145 and MCF‐7 cells. DU‐145 cells were chosen as these cells were shown to be resistant to the effect of IPA‐3 in our previous studies and because they have a high level of expression of PAK‐1, compared to other prostate cancer cells.^18^ MCF‐7 cells were chosen based on the fact that they had the highest level of PAK‐1 expression among the cells described in Figure 1. Immunoblot analysis demonstrated that transduction of both DU‐145 and MCF‐7 cells with shRNA lentiviral particles significantly reduced the expression of PAK‐1, as compared to cells transduced with the control shRNA lentiviral particles (Figure 2). The decrease in expression was rechecked and was maintained in cells for 6‐8 months (Supplemental Figure S2). These data show that stable DU‐145 PAK‐1 KD and MCF‐7 PAK‐1 KD cell lines were successfully established.

### 3.3 PAK‐1 knockdown altered cell morphology and growth

**Figure 3:**
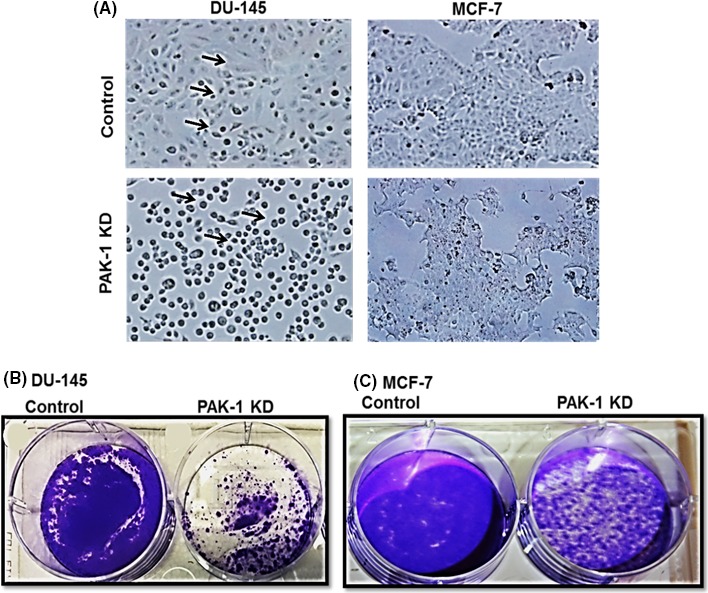
Effect of PAK‐1 Inhibition on prostate and breast cancer cell morphology and growth. (A) Effect of PAK‐1 shRNA on morphology of DU‐145 and MCF‐7 cells. (B‐C) Effect of PAK‐1 inhibition on cell proliferation in DU‐145 and MCF‐7 cells. Arrows refer to rounding up phenotype. Cells were stained using crystal violet. Data are representative of at least three different experiments using different passages (n = 3)

Inhibition of PAK‐1 expression correlated to changes in the morphology of DU‐145 cells as compared to their controls (Figure 3A). Inhibition of PAK‐1 in DU‐145 resulted in a more rounded cell type as opposed to the control cells (Figure 3A). Furthermore, these cells never reached full confluency, even after long incubations as opposed to control cells. MCF‐7 cells had a less prominent morphological change (Figure 3A). Besides, these cells seemed to be less adherent to the bottom of the culture flasks. Changes in cell morphology correlated to decreases in cell proliferation as assessed using crystal violet staining (Figure 3B,C).

### 3.4 PAK‐1 knockdown inhibited the migration and invasion of DU‐145 and MCF‐7 cells

**Figure 4:**
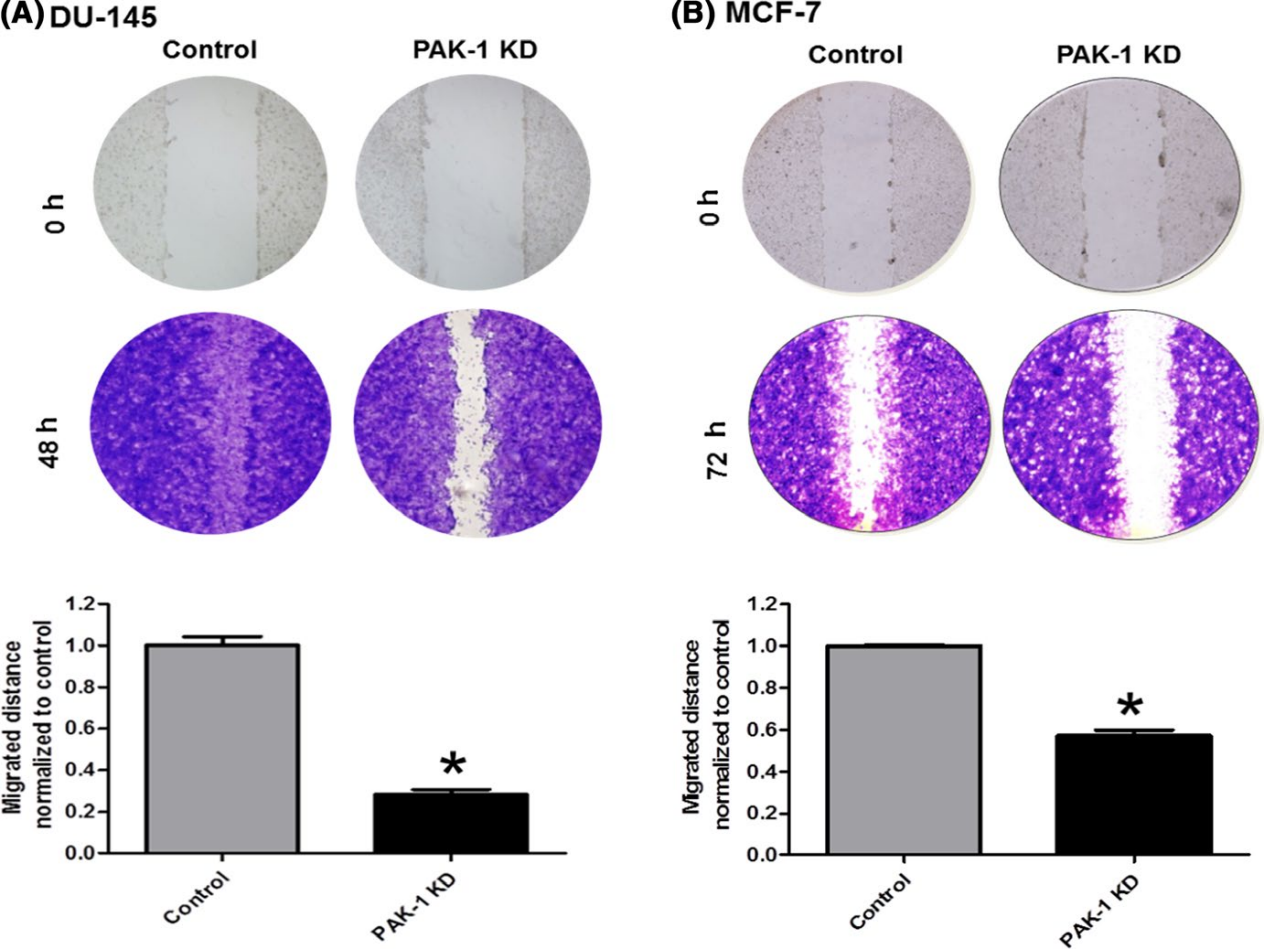
Effect of PAK‐1 inhibition on prostate and breast cancer cell migration. Cell wound closure assays in DU‐145 (A) and MCF‐7 (B) cells were used to determine the effect of PAK‐1 inhibition on cell migration. Data in A and B are representative of at least three (n = 3) different passages. Data are presented as the mean ± SEM; *Indicates a significant (*P* < .05) difference as compared to control cells

**Figure 5:**
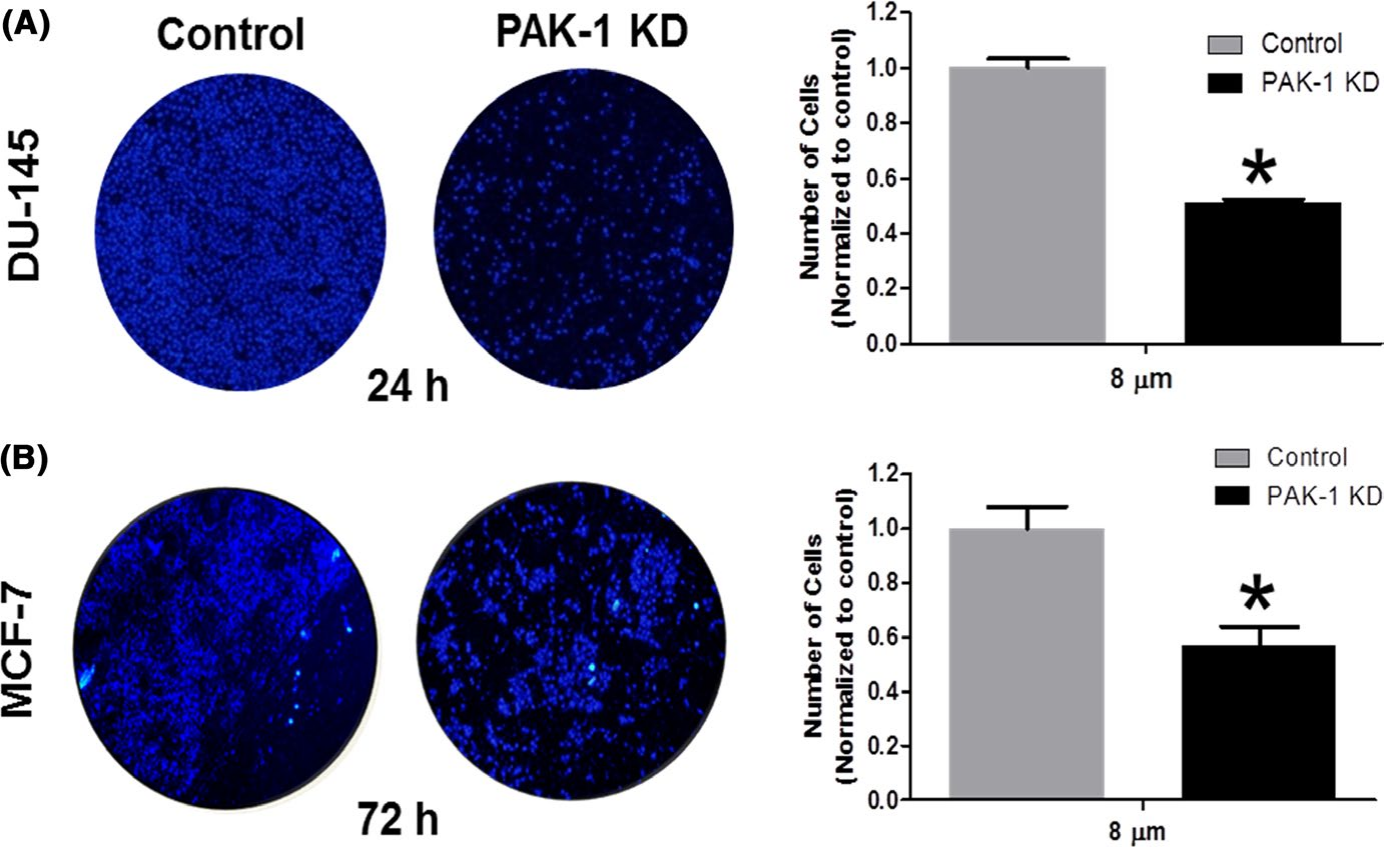
Effect of PAK‐1 inhibition on prostate and breast cancer cell invasion. Transwell^®^ migration assays were used to determine the effect of PAK‐1 knockdown on the ability of DU‐145 (A) and MCF‐7 (B) cells to migrate to the chemoattractant chamber after 24 and 72 hours. Cells were stained with DAPI and counted under a fluorescence microscope. Data are representative of three (n = 3) different experiments done on three different passages. Data are presented as the mean ± SEM; *Indicates a significant (*P* < .05) difference as compared to control cells

We used the scratch and Transwell^®^ assays to assess the role of PAK‐1 on cell migration and invasion. Inhibition of PAK‐1 expression decreased the wound closure in both cell types (Figure 4). Significant decreases in the gaps created by the scratch assay were seen in DU‐145 control cells after 48 hours, and after 72 hours in MCF‐7 control cells. The Transwell^®^ assay was used to differentiate between proliferation and migration, which are both represented in the wound healing assay. As expected, inhibition of PAK‐1 expression decreased migration in both DU‐145 and MCF‐7 cells as compared to control cells (Figure 5). These results support the hypothesis that PAK‐1 mediates the proliferation and migration of prostate and breast cancer cells *in vitro*.

### 3.5 PAK‐1 knockdown altered the expression of other proteins

**Figure 6:**
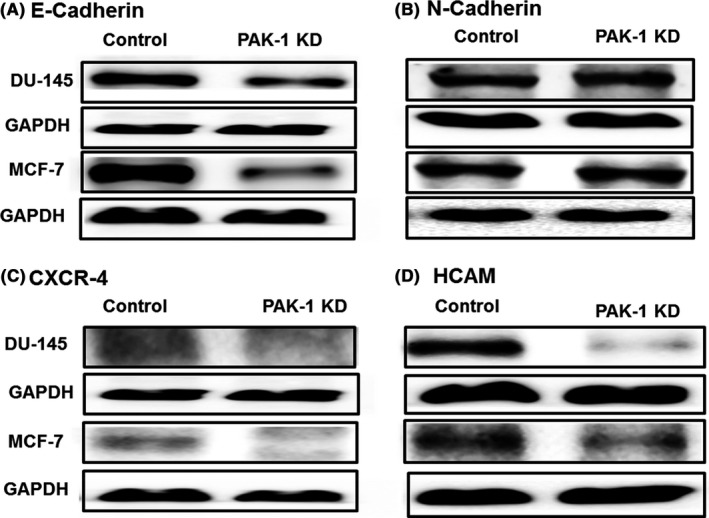
Effect of PAK‐1 inhibition on the expression of selected cancer‐related proteins. (A) E‐cadherin, (B) N‐cadherin, (C) CXCR‐4, and (D) HCAM. PAK‐1 expression was inhibited in DU‐145 and MCF‐7 cells using shRNA lentiviral particles and the effect of PAK‐1 KD on the expression levels of select proteins related to cancer cell proliferation was determined using immunoblot analysis. Data are representative of three (n = 3) different experiments

We further assessed the effect of PAK‐1 inhibition on DU‐145 and MCF‐7 cell growth by determining differences in the expression of E‐cadherin, N‐cadherin, CXCR‐4, and HCAM using immunoblot analysis (Figure 6). These proteins were chosen as many studies have shown their involvement in cancer proliferation and progression.^23^, ^24^, ^25^, ^26^ Specifically, E‐ and N‐cadherin have been shown to play an important role in EMT while CXCR4 plays an important role in cancer cell metastasis through chemoattraction. Our data show that the inhibition of PAK‐1 decreased the expression of E‐cadherin, HCAM, and CXCR‐4 in both cell lines (Figure 6 A, C, and D). In contrast, inhibition of PAK‐1 did not alter the expression of N‐cadherin, as compared to the respective control as shown in Figure 6B.

### 3.6 PAK‐1 inhibition altered the toxicity of IPA‐3 to DU‐145 and MCF‐7 cells

**Figure 7:**
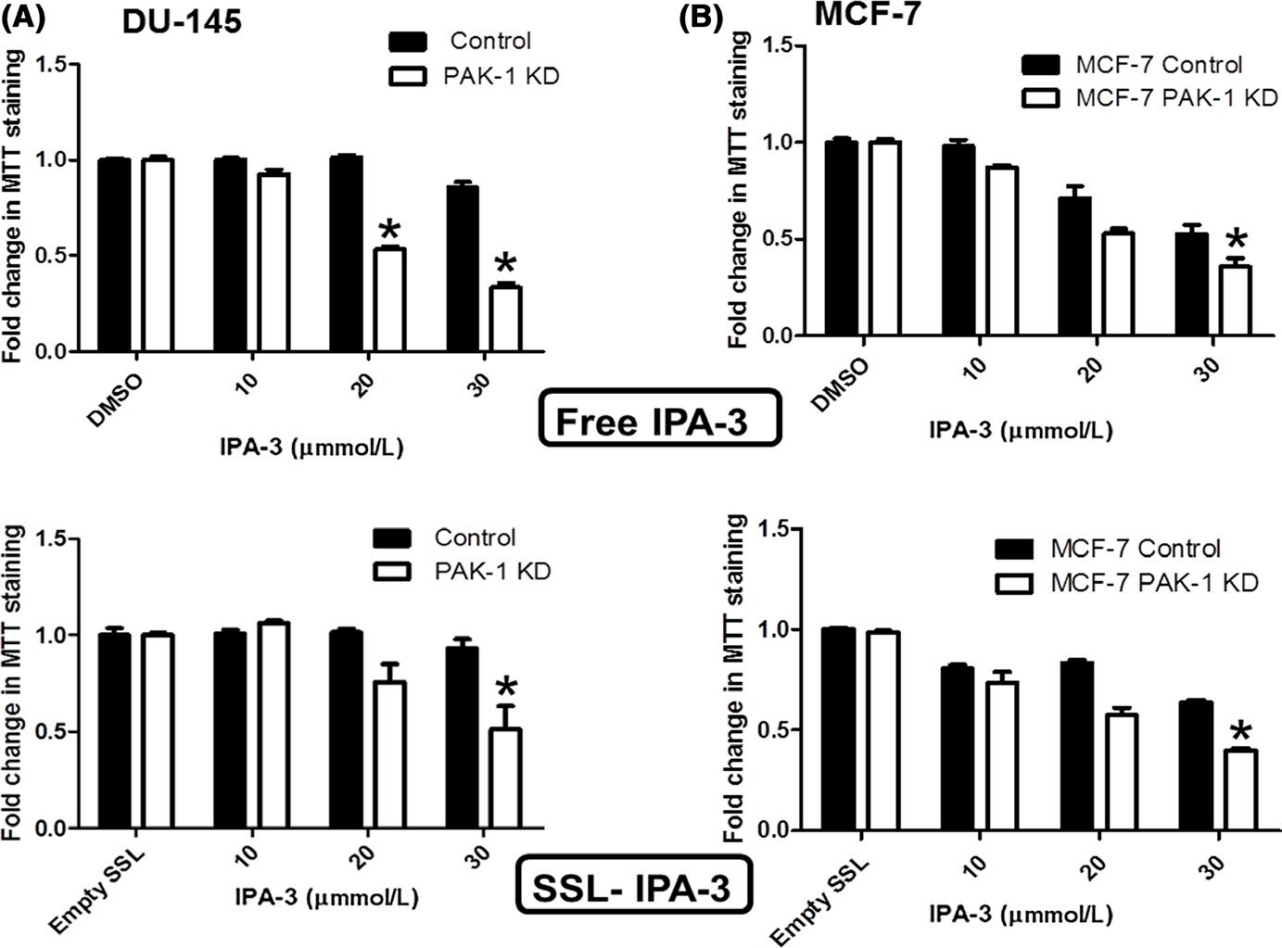
Effect of PAK‐1 inhibition on the susceptibility of DU‐145 and MCF‐7 cells to IPA‐3. Dose‐dependent effect of free IPA‐3 and SSL‐IPA‐3 on MTT staining in DU‐145 PAK‐1 KD (A) and MCF‐7 PAK‐1 KD (B), respectively, 48 hours after treatment. DMSO and empty liposomes were used as control vehicles for IPA‐3 and SSL‐IPA‐3, respectively. Data are representative of three (n = 3) different experiments done on three different passages. Data are presented as the mean ± SEM; *Indicates a significant (*P* < .05) difference as compared to control cells

We studied the effect of PAK‐1 inhibition on the activity of free IPA‐3 and IPA‐3 encapsulated in liposomes (SSL‐IPA‐3) in both DU‐145 and MCF‐7 cells using the MTT assay. As shown in Figure 7A, treatment of control DU‐145 cells with free IPA‐3 did not decrease MTT staining at any concentration studied as compared to cells treated with DMSO. In contrast, inhibition of PAK‐1 decreased MTT staining as compared to control cells. Similar results were seen with SSL‐IPA‐3 and in MCF‐7 cells (Figure 7B). The difference in susceptibility of DU‐145 and MCF‐7 to free IPA‐3 is similar to what we previously reported.^18^ These data support the hypothesis that inhibition of PAK‐1 in DU‐145 and MCF‐7 cells enhances their responsiveness to IPA‐3.

**Figure 8:**
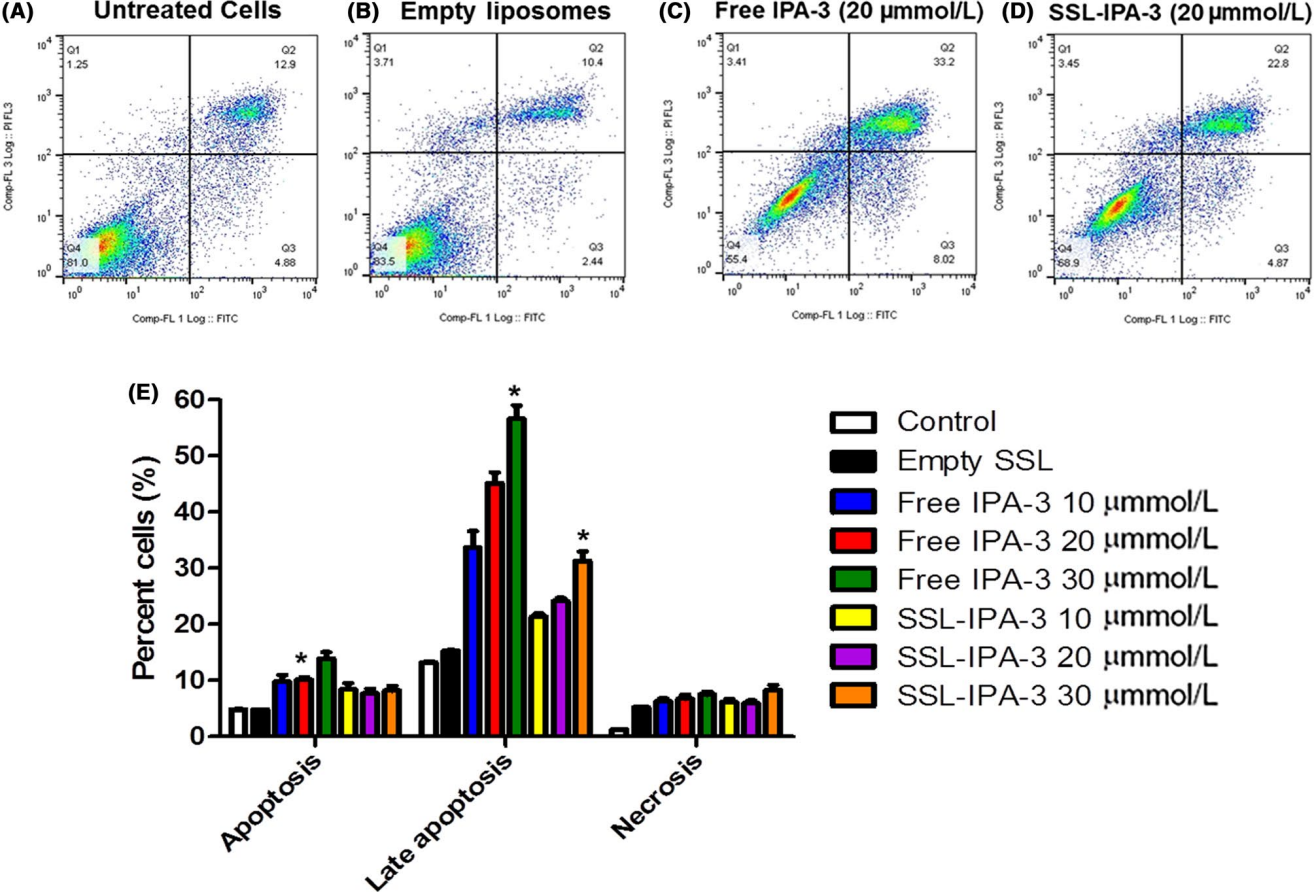
Effect of PAK‐1 inhibition on the efficacy of IPA‐3 and SSL‐IPA‐3 in DU‐145 PAK‐1 KD cells using annexin V and PI staining. (A‐D) Scatter plots demonstrating annexin V (x‐axis) and PI (y‐axis) staining in control untreated cells (A) and cells treated with empty liposomes (B), IPA‐3 treated (C) and SSL‐IPA‐3 treated (D) DU‐145 PAK‐1 KD cells for 48 hours. The quantification of staining is shown in (E). Data are representative of three (n = 3) different experiments done on three different passages. Data are presented as the mean ± SEM; *Indicates a significant (*P* < .05) difference as compared to control cells

**Figure 9:**
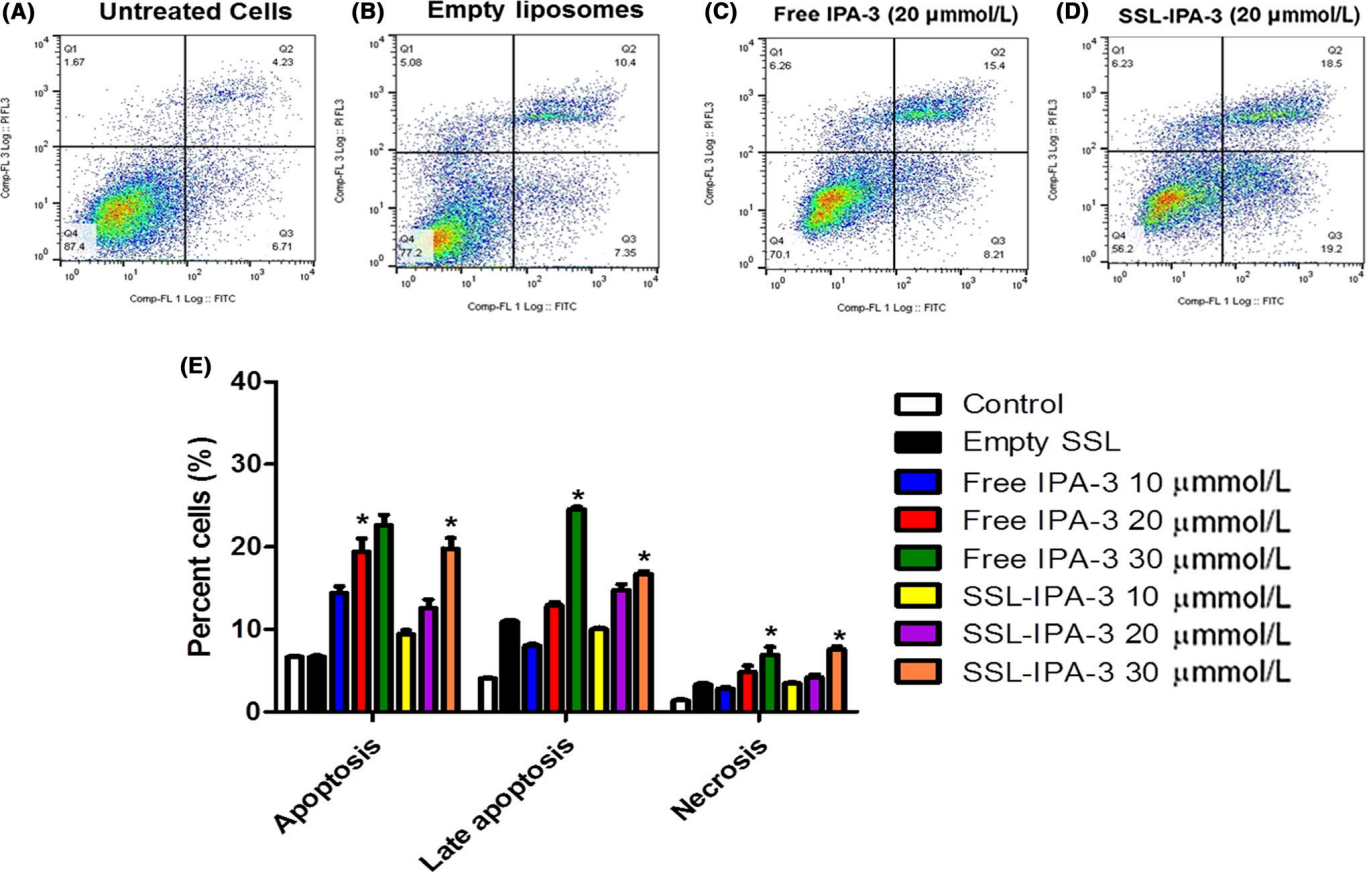
Effect of PAK‐1 inhibition on the efficacy of IPA‐3 and SSL‐IPA‐3 in MCF‐7 PAK‐1 KD cells using annexin V and PI staining. (A‐D) Scatter plots demonstrating annexin V (x‐axis) and PI (y‐axis) staining in control untreated cells (A) and cells treated with empty liposomes (B), IPA‐3 treated (C) and SSL‐IPA‐3 treated (D) MCF‐7 PAK‐1 KD cells for 48 hours. The quantification of staining is shown in (E). Data are representative of three (n = 3) different experiments done on three different passages. Data are presented as the mean ± SEM; *Indicates a significant (*P* < .05) difference as compared to control cells

Annexin V and PI staining were assessed using flow cytometry to investigate the mechanisms by which free IPA‐3 and SSL‐IPA‐3 induced death in DU‐145 PAK‐1 KD (Figure 8) and MCF‐7 PAK‐1 KD cells (Figure 9). In the untreated (control) samples, most of the cells (85%‐87%) stained negative for annexin V and PI, indicating that they were viable. In contrast, treatment of DU‐145 and MCF‐7 PAK‐1 KD cells with free IPA‐3 and SSL‐IPA‐3 resulted in concentration‐dependent increases in annexin V and PI staining at 48 hours. Treatment of cells with empty liposomes did not appreciably increase the percent cells staining positive for either annexin V or PI. Treatment of DU‐145 PAK‐1 KD and MCF‐7 PAK‐1 KD cells with free IPA‐3 resulted in concentration‐dependent increases in cells staining positive for annexin V, as well as those staining positive for both annexin V and PI. Similar results were seen when DU‐145 PAK‐1 KD and MCF‐7 PAK‐1 KD cells were exposed to SSL‐IPA‐3; however, the level of staining was not as high as that seen in free IPA‐3 treated cells. Increases in PI staining were only seen at the highest doses of IPA‐3 and SSL‐IPA‐3 used, suggesting that the primary mechanism of cell death was apoptosis.

## 4 DISCUSSION

PAK‐1, a serine/threonine kinase, was suggested to play a major role in various cancers.^27^ Studies have even suggested that PAK‐1 is a tumor oncogene and a therapeutic target for the treatment of cancer.^13^, ^15^ Compared to prostate cancer, there are limited studies on the role of PAK‐1 in other cancers. Studies that used mouse knockout models demonstrated a role of PAK‐1 in cell migration.^2^ Data from our study support that PAK‐1 mediated cell growth in several different cancer cell lines, including those derived from breast and melanoma. Our previous study also suggested that prostate cancer cells with the highest expression of PAK‐1 (DU‐145) as well as the breast cancer cells (MCF‐7) were the least susceptible to IPA‐3^18^^,^ raising the concern that the toxicity of IPA‐3 may not be dependent on PAK‐1 expression. We tested this possibility using a dual approach of pharmacological and molecular inhibition. As expected, both approaches decreased the growth of prostate and breast cancer cells. These data support the conclusion that toxicity of IPA‐3 to cancer cells is mediated in part by PAK‐1, and support the hypothesis that PAK‐1 acts as an oncogene in not only prostate cancer cells,^15^ but extends this hypothesis to breast cancer cells and melanoma.

Data from this study also demonstrated that PAK‐1 inhibition decreased the expression of E‐cadherin, CXCR‐4, and HCAM. E‐cadherin plays a crucial role in the epithelial adherens junctions, where several proteins interact, including α‐ and β‐catenin, to mediate the actin cytoskeleton.^28^ Inhibition of E‐cadherin expression is required for EMT and plays a role in cancer migration and metastasis.^29^ These data align with previous reports showing that PAK‐1 regulates cytoskeletal organization and cell–cell interactions.^30^ These data may also explain why PAK‐1 inhibition changes the morphology of DU‐145 cells. CXCR‐4 is a G‐protein‐coupled receptor that has been shown to be overexpressed in multiple cancers and is involved in cell adhesion, survival, and growth.^31^ Studies have shown that suppression of CXCR‐4 *in vitro* inhibited cell invasion.^32^ Our data agree with these findings and report the novel finding that PAK‐1 regulates the expression of CXCR‐4 in both DU‐145 and MCF‐7 cells. HCAM, also referred to as CD44 antigen, is a cell‐surface glycoprotein that has been shown to be involved in cell adhesion, cellular interactions, and migration and was suggested as a potential diagnostic and prognostic marker of malignancy in breast and ovarian cancers.^33^, ^34^, ^35^ Our data show that the expression of HCAM was inhibited following PAK‐1 inhibition. To our knowledge, this is the first report that PAK‐1 may mediate the expression of HCAM in any cell type.

PAK‐1 expression is increased during the early stages of human breast cancer progression.^12^, ^36^ Studies suggest that PAK‐1 overexpression can predict tumor recurrence and resistance to tamoxifen, which is a selective estrogen receptor modulator commonly used for the treatment of hormone‐receptor‐positive, early stage breast cancer.^37^ Toward this hypothesis, the IC_50_ of IPA‐3 was highest in estrogen receptor (ER)‐positive breast cancer cells, MCF‐7 and BT‐474, while the IC_50_ was lower in ER‐negative cells (MDA‐231 and MD‐468). MDA‐435 cells were originally identified as breast cancer, but are now believed to be melanoma in origin. However, these cells are also ER‐negative^38^ and had a relatively low IC_50_ as compared to ER‐positive cells. The higher IC_50_ in MCF‐10A is interesting given that these cells are ER‐negative, however, these are not cancer cells, and are considered models for normal breast cell function.

The overexpression of PAK‐1 is believed to be responsible for the phosphorylation of the estrogen receptor, creating promiscuous phosphorylated receptors resistant to tamoxifen treatment.^39^, ^40^, ^41^ Our studies further support the link between ER expression and PAK‐1 expression in breast cancer cells and suggest a link between the efficacy of PAK‐1 inhibitors and ER status.

We previously showed that IPA‐3, in both free and in liposomal forms, inhibits prostate cancer growth *in vitro* and *in vivo*.^18^ However, the efficacy of IPA‐3 correlated to the expression of PAK‐1, with cells that express higher levels of PAK‐1 demonstrating decreased susceptibility.^18^ This phenomenon extended to liposome‐encapsulated IPA‐3 in that SSL‐IPA‐3 did not decrease cell viability in DU‐145 cells in our previous studies at any dose.^18^ This is of concern because it suggests that these liposomal formulations may not be as efficacious in the clinic in high‐grade cancers that overexpress PAK‐1. This also suggests the possibility that the efficacy of both IPA‐3 and SSL‐IPA‐3 was not specific to PAK‐1. Data in this study definitely show that PAK‐1 KD alters the toxicity of cells to IPA‐3, however, the data do not unequivocally show that IPA‐3 toxicity is totally dependent on PAK‐1. It is possible that IPA‐3 may also inhibit other PAK’s, such as PAK‐2 at higher concentrations.^17^^,^ 42 ^,^ 43 The loss of PAK‐1 in MCF‐7 cells may have also altered the kinetics of inhibition. Further studies are needed to confirm this hypothesis.

The mechanisms mediating the increased sensitivity of prostate and breast cancer cells to SSL‐IPA‐3 during PAK‐1 inhibition are probably not drastically different than those mediating the toxicity of free IPA‐3. Most likely, the decrease in PAK‐1 is shifting the dose curve to the left, essentially increasing the potency of IPA‐3. This hypothesis is supported by the fact that PAK‐1 inhibition resulted in similar mechanisms of cell death (apoptosis) that was seen in free IPA‐3, as determined using flow cytometry. Alterations in annexin V and PI staining also confirm the morphological data and data derived from MTT staining.

While our data suggest that PAK‐1 is a promising potential therapeutic target for some cancers, the IC_50_ value of IPA‐3 (~15 µM) is not optimal for clinical translation. This is one reason that liposomal encapsulation was used for IPA‐3, which is typically limited by its stability. Nevertheless, the data also suggest that more studies are needed to develop more potent PAK‐1 inhibitors. Such studies are already under way in our laboratory.

In summary, our data show that the pharmacological effect of IPA‐3 is mediated, in part, by PAK‐1, demonstrate some of the first data suggesting that IPA‐3 is a potential therapeutic treatment for breast cancer and melanoma, and demonstrate the efficacy of liposome‐encapsulated IPA‐3 in breast cancer cells. This is, as far as we know, the first report of a direct correlation between PAK‐1 expression and efficacy of IPA‐3 in breast cancer.

## DISCLOSURES

The authors declare no conflict of interests.

## AUTHOR CONTRIBUTIONS

Participated in research design: Najahi‐Missaoui, Somanath, and Cummings; Conducted experiments: Missaoui, Quach, Jenkins, and Dabke; Performed data analysis: Missaoui, Cummings, Somanath, Quach, Jenkins, and Dabke; Wrote or contributed to the writing of the manuscript: Missaoui, Quach, Cummings, and Somanath.

## Data Availability

The data that support the findings of this study are available from the corresponding author upon reasonable request.
