# Supplementary material for: Effect of P21‐activated kinase 1 (PAK‐1) inhibition on cancer cell growth, migration, and invasion
Source: Pharmacol Res Perspect. 2019 Sep 6;7(5):e00518. doi: 10.1002/prp2.518 (PMC6728842; doi:10.1002/prp2.518)
Supplement: Supplementary file 1 [file PRP2-7-e00518-s001.docx]

**
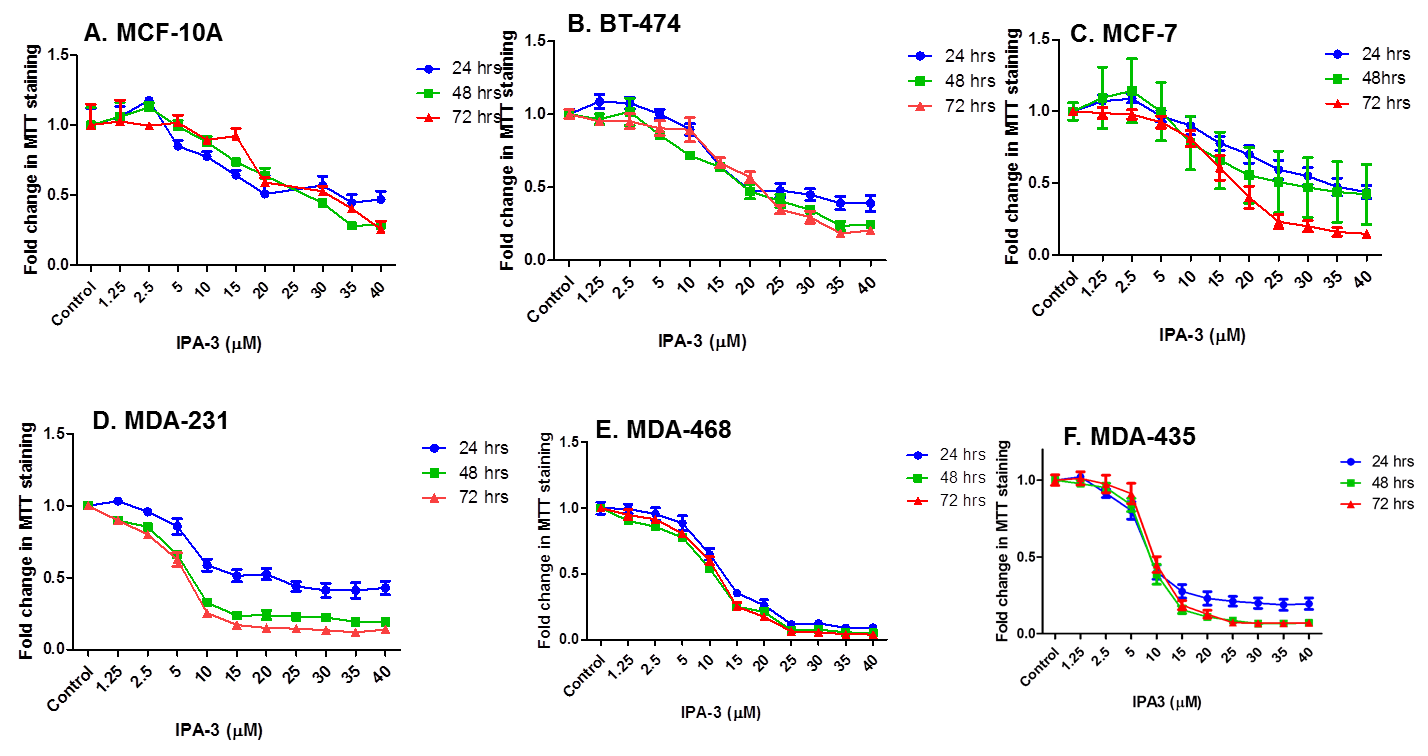
**

**Supplemental Figure S.1.** Effect of PAK-1 inhibitor IPA-3 on MTT staining in breast cancer and melanoma (MDA-435) cells. **(A-F)** Dose and time-dependent effects of free IPA-3 on MTT staining in the human breast transformed MCF-10A cells (**A**), breast cancer BT-474 (**B**), MCF-7 (**C**), MDA-231(**D**), MDA-468(**E**) and melanoma MDA-435 (**F**) cells respectively 24, 48 and 72 hrs after treatment (n = 3). Data are presented as the mean ± SEM of the fold change in MTT staining as a function of IPA-3 concentration.

**
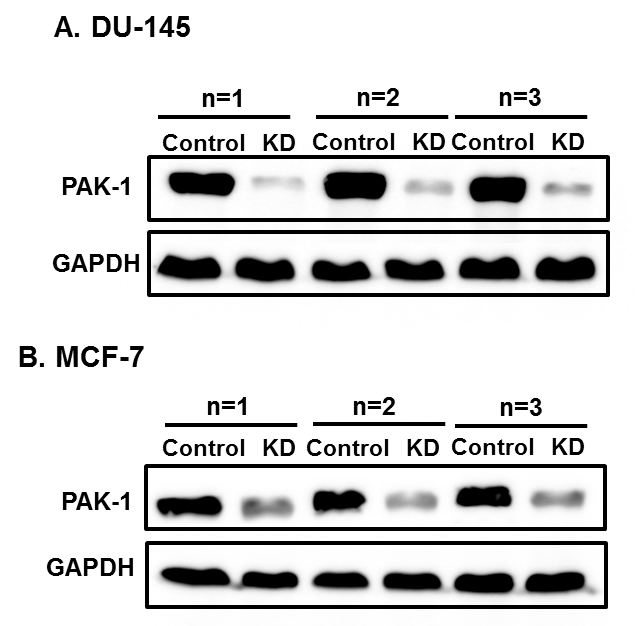
**

**Supplemental Figure S.2.** Long-term effect of PAK-1 knockdown on its expression in prostate and breast cancer cells. Expression of PAK-1 in DU-145 PAK-1 KD (**A**) and MCF-7 PAK-1 KD (**B**) cells compared to their respective controls (determined 6-8 months after transfection) by immunoblot analysis. Data are representative of 3 (n = 3) different cell passages.
