# Supplementary figures and images for: Effect of P21‐activated kinase 1 (PAK‐1) inhibition on cancer cell growth, migration, and invasion
Source: Pharmacol Res Perspect. 2019 Sep 6;7(5):e00518. doi: 10.1002/prp2.518 (PMC6728842; doi:10.1002/prp2.518)

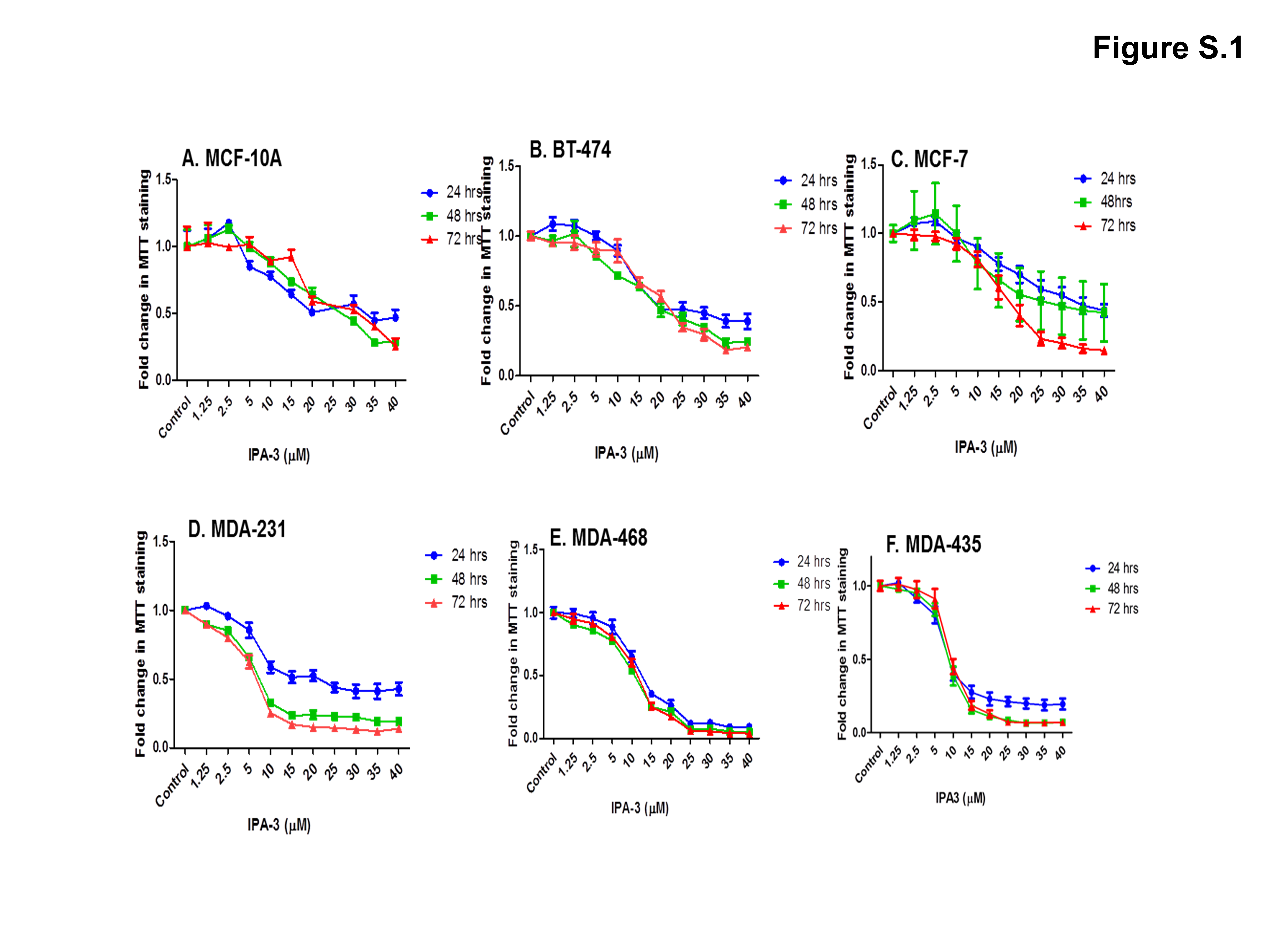

Supplement: Supplementary file 2 [file PRP2-7-e00518-s002.TIF]

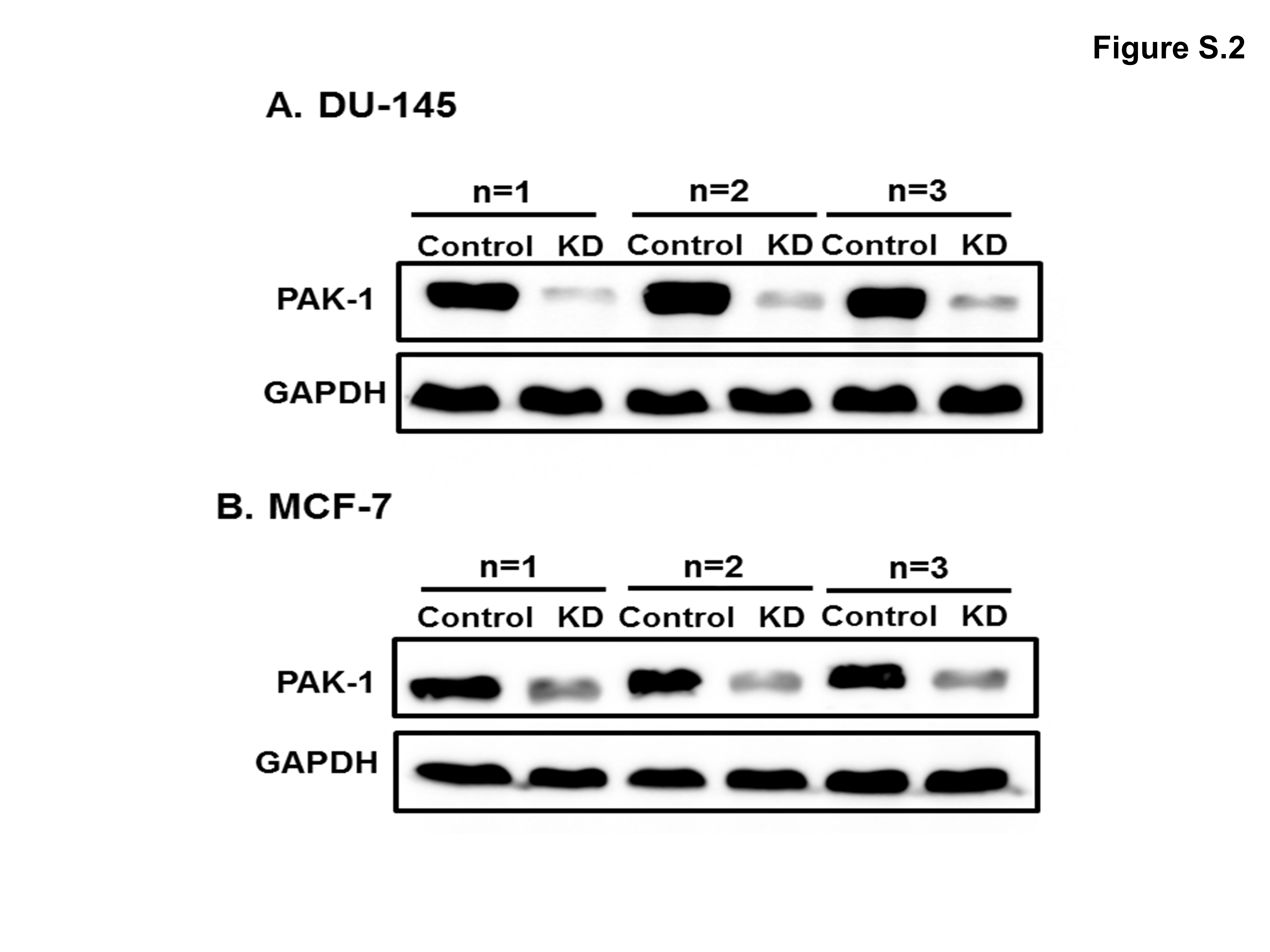

Supplement: Supplementary file 3 [file PRP2-7-e00518-s003.TIF]
